# Abiotic Stresses Intervene with ABA Signaling to Induce Destructive Metabolic Pathways Leading to Death: Premature Leaf Senescence in Plants

**DOI:** 10.3390/ijms20020256

**Published:** 2019-01-10

**Authors:** Muhammad Asad Ullah Asad, Shamsu Ado Zakari, Qian Zhao, Lujian Zhou, Yu Ye, Fangmin Cheng

**Affiliations:** 1 Institute of Crop Science, College of Agriculture and Biotechnology, Zhejiang University, Hangzhou 310058, China; asad.gu@hotmail.com (M.A.U.A.); shamsuado@yahoo.com (S.A.Z.); qzh@zju.edu.cn (Q.Z.); 11816073@zju.edu.cn (L.Z.); 21716029@zju.edu.cn (Y.Y.); 2 Jiangsu Collaborative Innovation Centre for Modern Crop Production, Nanjing 210000, China

**Keywords:** premature leaf senescence, ABA biosynthesis, ABA signaling receptors, chlorophyll degradation, ABA-induced transcription factors

## Abstract

Abiotic stresses trigger premature leaf senescence by affecting some endogenous factors, which is an important limitation for plant growth and grain yield. Among these endogenous factors that regulate leaf senescence, abscisic acid (ABA) works as a link between the oxidase damage of cellular structure and signal molecules responding to abiotic stress during leaf senescence. Considering the importance of ABA, we collect the latest findings related to ABA biosynthesis, ABA signaling, and its inhibitory effect on chloroplast structure destruction, chlorophyll (Chl) degradation, and photosynthesis reduction. Post-translational changes in leaf senescence end with the exhaustion of nutrients, yellowing of leaves, and death of senescent tissues. In this article, we review the literature on the ABA-inducing leaf senescence mechanism in rice and *Arabidopsis* starting from ABA synthesis, transport, signaling receptors, and catabolism. We also predict the future outcomes of investigations related to other plants. Before changes in translation occur, ABA signaling that mediates the expression of *NYC*, *bZIP*, and *WRKY* transcription factors (TFs) has been investigated to explain the inducing effect on senescence-associated genes. Various factors related to calcium signaling, reactive oxygen species (ROS) production, and protein degradation are elaborated, and research gaps and potential prospects are presented. Examples of gene mutation conferring the delay or induction of leaf senescence are also described, and they may be helpful in understanding the inhibitory effect of abiotic stresses and effective measures to tolerate, minimize, or resist their inducing effect on leaf senescence.

## 1. Introduction

Leaf senescence is an age-dependent programmed cell death that propels nutrients from aging cells to developing and storage tissues and causes the exhausted materials to accumulate in dying cells [1,2]. The first span is to end the transition of the reproductive cycle, which involves a series of complex metabolic signals and regulatory factors to induce post-transcriptional changes prior to physiological and metabolic changes that lead to the onset of leaf senescence. The earliest and most significant change in cell structures is the breakdown of the chloroplast, an organelle that contains up to 70% of the leaf protein. Metabolically, carbon assimilation is replaced by the catabolism of chlorophyll (Chl) and macromolecules such as proteins, membrane lipids, and RNA [3,4]. The increased catabolic activity is responsible for converting the cellular materials of the growth phase of leaves into exportable nutrients that are supplied to developing seeds or other growing organs. This natural leaf senescence is responsible for the continuity of life by transferring nutrients and genetic information from generation to generation [5].

Abiotic stresses and biotic factors integrate with phytohormone regulatory factors (i.e., abscisic acid (ABA), ethylene, silicic acid, jasmonic acid) and aging factors to alter plant growth and development [6]. Plants use their phytohormone regulatory network to alleviate the effects of environmental stresses as a survival mechanism. In the interplay among plant development, stress responses, and phytohormones, ABA plays a pivotal role in the coordinated regulation of many physiological processes in an age-respective manner ranging from seed germination to maturation [7,8,9]. ABA constitutes a homeostasis system during stress, and this system includes interactions with the ubiquitin–proteasome system to alter the degradation of transcriptional regulators [10], the stomatal closure that restricts cellular growth [11], and the induction of senescence-associated genes (SAGs), non-yellow coloring 1 (*NYC1*), *SGR*, *PPH*, and pheophorbide a oxygenase (*PAO*) gene expressions [12]. The levels of endogenous ABA and genes associated with ABA signaling increase in senescent leaf tissues [13]. In *Arabidopsis At4G25080*, *At5G54190*, *At1G44446*, *At3G51820*, PAO-encoded magnesium protoporphyrin IX methyltransferase, protochlorophyllide reductase, chlorophyllide-a oxygenase and bacteriochlorophyll a synthase affect Chl synthesis and leaf development [14,15,16,17,18]. In rice, pentatricopeptide repeat genes regulate the expression of chloroplast genes, resulting in leaf senescence [19], while exogenous ABA has been reported to increase the expression of SAGs [20,21,22].

ABA triggers metabolic changes to induce leaf senescence in old leaves for the survival mechanism of sprouting leaves. However, once stress overwhelms the homeostasis mechanism, leaf senescence dominates to induce premature leaf senescence, which may reduce yield, and premature leaf senescence may serve as a pruning limiting factor to enhance productivity in modern agriculture. Within plants, the consolidated structure of endogenous ABA depends on ABA biosynthesis, transport, ABA signaling, and catabolism, which may constitute the central role leading routine leaf senescence. ABA coordination with dominating environmental stresses causes premature leaf senescence, which consequently reduces yield in crops by limiting the growth phase. Thus, information about the mechanism of ABA-mediated leaf senescence not only enhances our understanding of a fundamental biological process but also possibly provides a means to control leaf senescence for the improvement of crop yield. ABA synthesis and propagation have been reviewed already [23,24], but the complete crosstalk between ABA and leaf senescence should be elaborated. In this paper, we discuss the ABA core pathways for the induction of leaf senescence, starting from ABA biosynthesis and catabolism, ABA transport, and ABA signaling receptors. Then, the integrated mechanism of abiotic factors with ABA for the induction of premature leaf senescence, covering the chloroplast degradation and photosynthesis decline, ROS generation and oxidative stress, kinase protein regulation, and secondary messenger Ca^2+^ are discussed. Pre-translational changes such as the activation of TFs (NACs, bZIPs, WRKYs) are explained to regulate the expression of senescence associated genes (SAGs) in *Arabidopsis* and rice, and the future prospects of TFs related with other plants are proposed. Post-translational factors of membrane-associated proteins and target of rapamycin during leaf senescence are also discussed. The modifications of genes involved in the regulation of plant responses to ABA and potential prospects regarding the improvement of plant regulation are also described. Our review covers recent findings about the interaction of ABA with carbon and sugar signaling as well as ethylene to induce leaf senescence.

## 2. ABA Core Pathways for the Regulation of Leaf Senescence

### 2.1. ABA Biosynthesis and Catabolism

**Figure 1:**
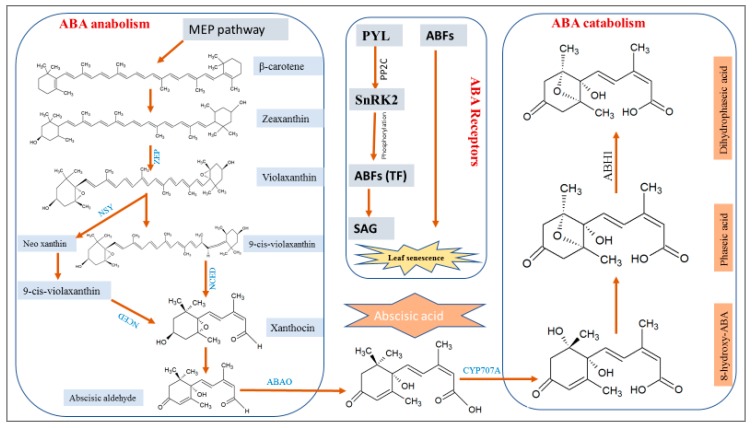
Abscisic acid (ABA) biosynthesis, ABA catabolism, and ABA signaling sensors (receptors) pathways. SAG: senescence-associated gene, In MEP (Methyl Erythritol Phosphate), β-carotene is converted into zeaxanthin, ZFP (zeaxanthin epoxidase) catalyze zeaxanthin into violaxanthin, NSY (neo xanthin synthase) convert violaxanthin into neo xanthin and 9 cis-violaxanthin, NCED (9-cis-epoxycarotenoid dioxygenase) convert 9 cis-violaxanthin into xanthocin and then abscisic aldehyde, ABAO: abscisic aldehyde oxidase catalyzes abscisic aldehyde into abscisic acid. ABA is sensed by ABA receptors; PYL (pyrabactin resistance 1-like), PP2C (protein phosphatase 2C) which activate SnRK2. The activated SnRK2 phosphorylate ABFs (ABA-responsive element-binding factors) to induce the expression of SAGs (senescence-associated genes).

Genes related to ABA de novo biosynthesis, ABA receptors, and downstream signaling relays have been characterized in *Arabidopsis* and rice [25]. The first process is to send signals to ABA production by abiotic stresses in regulatory feedback mechanisms [26,27,28]. ABA biosynthesis is controlled by two pathways, namely, a direct pathway in fungi and an indirect pathway in plants. ABA is produced by an indirect carotenoid pathway, which comprises three stages. First, zeaxanthin is produced in plastids by zeaxanthin epoxidase (ZEP) [29]. Second, zeaxanthin is converted into 9-cis-violaxanthin and 9-cis-neoxanthin by neoxanthin synthase, which is encoded by AtABA4 [30]. They (i.e., 9-cis-violaxanthin and 9-cis-neoxanthin) are converted to xanthoxin (Xan) by 9-cis-epoxycarotenoid dioxygenase (NCED), which is a rate-limiting reaction in ABA biosynthesis [29,31,32]. Third, Xan is transferred to the cytosol and converted to abscisic aldehyde (ABAld) by xanthoxin dehydrogenase [33,34]. Then, ABAld is catalyzed into ABA by abscisic aldehyde oxidase (ABAO), as shown in Figure 1 [35]. The catalytic steps of ABA biosynthesis are mediated by ABA4, ABA1/LOS6, NCED, ABA3/LOS5, and ABA4, which convert β-carotene to ABA [6,7].

In rice, mutants with altered carotenoid precursor biosynthesis, such as flawed in carotenoid isomerase, phytoene desaturase, lycopene b-cyclase, and z-carotene desaturase, have also been characterized. In maize, 10 mutants with blocked carotenoid precursors of ABA synthesis (i.e., vp2, vp5, vp7, vp9, w3, y3, …, y9) have been identified [36,37]. However, the direct mechanism of explained leaf senescence with the direct impact of these hormones should be further elaborated.

ABA concentration varied greatly among different plant tissues and growth stages. Abiotic stresses increase ABA concentration, as the fastest response to restrict cellular growth [6,38]. If the rate of ABA catabolism is higher than that of ABA anabolism, then the inducing capacity of ABA catabolism decreases. The stress recovery signal transduces ABA catabolism with 8’ hydroxylation of ABA by the CYP707A family. This catalytic route leads to an unstable intermediate 8’-hydroxy-ABA, which soon changes its stable isomer, phaseic acid (PA) [39,40]. Later, PA is hydrated into dihyrdrophaseic acid by ABH1 (Figure 1).

### 2.2. ABA Transport

ABA is usually produced at the site of action, and is seldom transported long distances during leaf senescence [9]. ABA transport is controlled by transporter families to perceive ABA at the target site [41]. In the crosstalk related to ABA transport, it should be determined whether ABA produced in roots is transported to aging or targeted leaves prone to leaf senescence or if ABA is only produced at the site of action. In cells, ABA is transported by diffusion due to its selective cell membrane permeability [24]. ABA transporter genes, namely, *AtMRP5* of the ABC transporter family and an ATP-binding cassette transporter, have been identified to be involved in hormone signaling and water use [42,43]. In short-distance movement, *PDR12*/*ABCG40* and *AtABCG25* are involved in ABA movement [44]. *AtABCG25* is expressed in the membrane of vascular tissues at the site of ABA synthesis, which creates membrane vesicles to transport ABA through the intercellular signaling pathway in which *AtABCG22* encodes an ABA importer [45]. The mechanism of long-distance movement based on a critical signal messenger is complex and dependent on pH gradient.

### 2.3. ABA Signaling Receptors

ABA receptors are the first important molecular components for the relay of ABA-mediated leaf senescence signaling. ABA is sensed by pyrabactin resistance 1 (PYR1) and pyrabactin resistance 1-like (PYL)/regulatory component of ABA receptor proteins (RCAR), protein phosphatase 2C (PP2C), and SNF1 (sucrose non-fermenting)-related protein kinase 2 (SnRK2) [46,47,48]. In *Arabidopsis*, 14 AtPYLs have been studied at molecular, biochemical, and structural levels related to stress signaling [49,50]. These PYLs prevent clade A protein PP2Cs from inhibiting SnRK2s, which regulates the expression of phosphorylated transcription factors (ABA-responsive element-binding factors; ABFs) to induce ABA-responsive SAGs, creating the yellowing symptoms of leaf senescence [51]. Therefore, PYLs play a functional role in regulating stress responses mediated by ABA (Figure 1) [52]. *OsPYL3* from a deep-rooted, drought- and heat-tolerant upland *Indica* rice cultivar Nagina-22 (N22) has been isolated and compared using *PYL3* expression in N22 to a drought-susceptible rice genotype IR64 in response to various abiotic stresses. A receptor-like kinase 1 (ABF) is the positive regulator and upstream component of ABA signaling during senescence. Kinase 1 is the membrane-bound leucine-rich receptor with increased expression in leaf senescence with ABA. Accelerated leaf senescence is observed in *RPK1*-overexpressing mutants [53,54]. The functional characterization of these ABA receptors with respect to mediating the response to diverse endogenous and environmental stresses is a subject of debate.

## 3. Integrated Mechanism of ABA-Induced Leaf Senescence

### 3.1. Relationship of ABA-Induced Leaf Senescence with Chloroplast Degradation and Photosynthesis Decline

The destruction of chloroplast structure, the degradation of Chl, and the reduction of photosynthesis ability are the first processes of leaf senescence, starting with Chl degradation and chloroplast destruction. Chl degradation is catalyzed by the *NYC* gene family in many plants [1,5,55]. The initial step of Chl degradation begins with the upregulation of *NYC1* encoding Chl *b* reductase that catalyzes Chl degradation, which induces Chl catabolism to convert Chl *b* into 7-hydroxymethyl-Chl *a*. It is catalyzed by two Chl *b* reductase compounds, namely, NYC1 and NYC1-like (NOL) [56,57]. Most Chl *b* molecules are in the light-harvesting antennae around photosystem II. Therefore, LpNYC1 proteins are possibly attached to the thylakoid membrane system in direct contact with or in close distance to Chl *b* molecules. *NYC1* and *NOL* null mutants lack two of their functional Chl *b* reductases (*Oryza sativa*) that show a stay-green phenotype. The stay-green phenotype of *NYC1*/*NOL* double mutant is stronger than that of each mutant alone [58]. Furthermore, delayed leaf senescence lacking *NYC1* null mutant predicts that *NYC1* expression is regulated by ABA and ethylene.

NYC1 and NOL proteins physically interact with each other and co-catalyze Chl degradation [56,59]. The key *cis*-elements in *NYC1* promoter, namely, ABA-response element (ABRE) (ACGTG), ACGT, GCCcore (GCCGCC), and *ethylene-inducible 3* (*EIN3*)/*EIL1*-binding sequence (T[TAG][GA]CGT[GA][TCA][TAG]), can be targeted by *ABA insensitive* 3 (*ABI3*), *ABI5*, and *ABF2*, 3, 4 in the ABA-signaling pathway [60,61]. GCCGCC and *EIN3*/*EIL1*-binding sequence (T[TAG][GA]CGT[GA][TCA][TAG]) are induced by ethylene-inducible TF and *EIN3*/*EIL1* in the ethylene signaling pathway [61]. Therefore, ABA signaling is crucial for Chl *b* reductase activities to catalyze the Chl degradation, the first part of leaf senescence.

In *Arabidopsis*, only *NYC1* mutants show the stay-green phenotype with significantly high Chl *b* content because of the overexpression of *LpNYC1*, suggesting *LpNYC1* as the functional orthologous *NYC1* gene. NYC1 protein (LpNYC1) is subcellularly localized in chloroplasts, with three putative transmembrane domains. Interestingly, *Arabidopsis NOL* mutants do not show the stay-green phenotype [62], indicating that the physiological role of the *NOL* homolog in Chl degradation is slightly different between *Arabidopsis* and rice.

**Figure 2:**
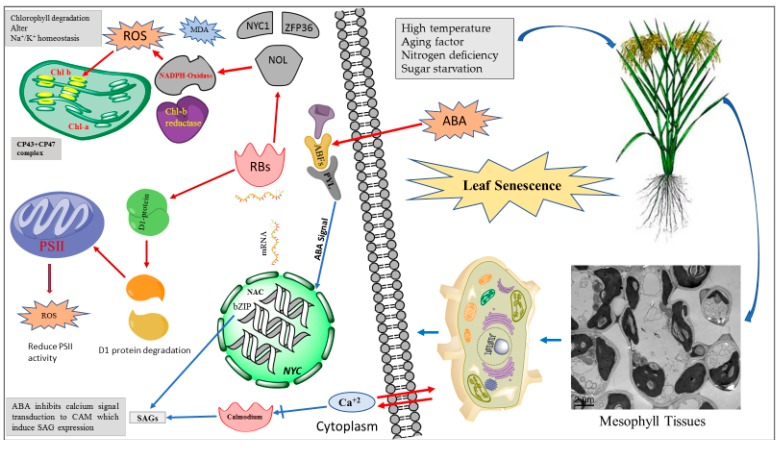
A schematic diagram of ABA signal transduction toward chlorophyll degradation and reduced photosynthetic activity. (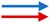 indicate induction and 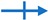 indicates suppression). Abiotic stresses induce ABA production, which is sensed by ABA signaling receptors (ABF, PYL). ABA signal induce expression of NAC, bZIP and NYC TFs. The activated TFs induce expression of SAGs which are translated in RBs (ribosomes) to synthesize (NOL, NADPH oxidase, Chl-*b* reductase). NDPH oxidase increase ROS production and Chl *b* reductase reduce PSII (photosystem II) efficiency and induce the degradation of D1 protein.

Another important attribute is the destruction of the chloroplast structure as the first sign before any visible change or deterioration, which leads to the decline of photosynthesis [63]. Photosynthesis reduction causes leaf yellowing as the startup of photosynthesis [64]. At the start of senescence, hydrophilic-associated membranes in the chloroplast degrade, and proteinases then catalyze other vascular organelles [63]. Photosystem II (PSII) controls electron absorption, transmission, and transformation for light reaction in the thylakoid membranes of the chloroplast [65]. Chl *a* in PSII is bound by CP43 and CP47 from the peripheral light-harvesting complex and transmits light energy to the reaction center; D1 subunits also form a heterodimer [66]. Excessive light damages the reaction center-binding protein D1 by producing ROS and reducing PSII activity [67]. A mechanism removes D1 protein controlled by FtsH proteases in the chloroplast as newly produced D1 protein is inserted into the thylakoid membrane [68]. However, under abiotic stresses, the repairing mechanism of PSII is disturbed because of the damage and inhibition of D1 protein [66]. In light-dependent leaf senescence, ABA triggers photodamage by degrading D1 protein in the PSII reaction center in senescing leaves. Partial shading leads to the slight accumulation of ABA in shaded areas, thus concomitantly delaying leaf senescence in shady areas [9]. These areas display low photoinhibition and oxidative damage with low O^−2^ and H_2_O_2_ accumulations, which induce the inhibition of D1 protein degradation and the accelerated D1 protein synthesis with low ABA. By contrast, unshaded areas show high ABA with increased photoinhibition. The decreased amount of D1 protein is found in non-shady areas and in exogenous ABA treatment to detached leaves. ABA induces the decreased expression of D1 encoding gene *PsbA* in senescing leaves to decrease D1 protein. Genes encoding D1 degradation (*FtsH1*, *FtsH2*, *FtsH3*, ... *FtsH8*) are sharply upregulated, creating a tendency for high degradation of D1 protein. Thus, sunlight interact with ABA to induce leaf senescence with decreased D1 protein synthesis and increased D1 degradation. Darkness leads to the decrease of ABA concentration in rice leaves along with the recovery of D1 protein, which consequently results in the re-synthesis of D1 protein and repair of PSII in light exposure (Figure 2).

### 3.2. Relationship of ABA-Induced Leaf Senescence with ROS Generation and Oxidative Stress

The antioxidative system works as the first shield against oxidative damage by regulating biological processes and responding to diverse environmental stimuli and aging factors. The stimulation of antioxidant defense systems induces NADPH oxidase to catalyze ROS production in plant tissues [69,70,71]. ABA mediates NADPH oxidase to start ROS generation, which act as rate-limiting second messengers in ABA signaling, consequently inducing leaf senescence in plants [72]. NADPH oxidase is encoded by nine *NOX* genes, and each *NOX* exhibits unique stress response characteristics. The transcripts of *NOX* isoform genes are affected by endogenous and exogenous ABA levels [21,72]. Exogenous ABA has been reported to stimulate *NOX* activities (expression levels of *OsNox2*, *OsNox5*, *OsNox6*, and *OsNox7*) to produce ROS in plant guard cells in response to ABA [70,73]. Among them, the expression levels of *OsNox5* and *OsNox7* are respectively dependent on low and high ABA concentrations, suggesting the strong association of *OsNox5* and *OsNox7* with distinct ABA concentrations in plant tissues [21]. NADPH is also required for O^2−^ production during ABA signal transduction, where *OsNox5* and *OsNox7* play a complementary role in detecting changes in ABA and in inducing O^2−^ production at distinct ABA levels during leaf senescence [21].

ZFP36, a novel rice C2H2-type zinc finger protein, is involved in ABA-induced antioxidant defense by regulating the expression of OsrbohE (*OsNox6*) and OsrbohB (*OsNox7*), indicating *OsNox6* and *OsNox7* as essential elements for ABA signaling [74]. ABA-mediated NADPH oxidases interact with ROS to stimulate the closing of stomata and activate plasma membrane Ca^2+^ channels in leaf guard cells [75]. In *Arabidopsis*, *AtrbohD* and *AtrbohF* encoding NADPH oxidases regulate Na^+^/K^+^ homeostasis and improve salt tolerance [71,76]. NtrbohD is necessary in ABA-induced H_2_O_2_ accumulation to improve resistance against various stresses [21]. In maize (*Zea mays*), *ZmrbohA*, *ZmrbohB*, *ZmrbohC*, and *ZmrbohD* are responsible for the biphasic response of ROS generation in ABA signal transduction [77]. NADPH oxidase in the plasma membrane is involved in ABA-induced antioxidant defense in leaves of maize seedlings [78]. NADPH inhibitors (DPI and IMD) suppress ABA-induced H_2_O_2_ production, lipid peroxidation, and delayed senescence in rice leaves [70,75,79]. DMTU, a chemical trap for H_2_O_2_, demonstrates the same results, indicating that ABA regulates H_2_O_2_ production. Furthermore, the increase in H_2_O_2_ content by ABA precedes the occurrence of leaf senescence and the increase in malondialdehyde (MDA) content. The mutant generated by ethyl methane sulfonate exhibits a remarkable increase in malondialdehyde content, a decrease in Chl content, a reduction in the number of chloroplasts and grana thylakoid, and modification of photosynthetic ability with an altered ability of ROS-scavenging enzymes (antioxidants) [80]. However, the regulatory mechanism of NADPH oxidase involved in ROS generation and ABA signaling during leaf senescence should be further investigated.

In most species, the distinctive feature of plant senescence is the increase in ROS and ABA hormone levels, accompanied by changes in enzyme activities related to ROS production and scavenging [21,81]. ABA causes oxidative stress to promote the senescence of rice leaves mediated by oxidative stress. In addition to the increase in H_2_O_2_, superoxide dismutase (SOD), ascorbate peroxidase (APOD), glutathione reductase (GR), and catalase (CAT) contents, a decrease in ascorbic acid (AsA) and glutathione (GSH) contents occurs in rice leaves [78]. Consequently, leading protein loss (senescence) and lipid peroxidation are observed in ABA-treated rice leaves [78]. Lipid peroxidation is considered as an important mechanism of leaf senescence [82]. Active oxygen species (AOS) can initiate lipid peroxidation [83]. ABA causes AOS generation, including H_2_O_2_ [84,85,86] and lipid peroxidation expressed as MDA production in plant cells [87]. Thus, ABA leads to oxidative stress in plant cells. ABA modulates abiotic stresses in coordination with other hormones and regulatory factors to induce cell death mechanism. Enhancing ABA increases susceptible diseases and pathogen attacks [88].

### 3.3. ABA Modulates Leaf Senescence by Activating Regulating Kinase Protein

**Figure 3:**
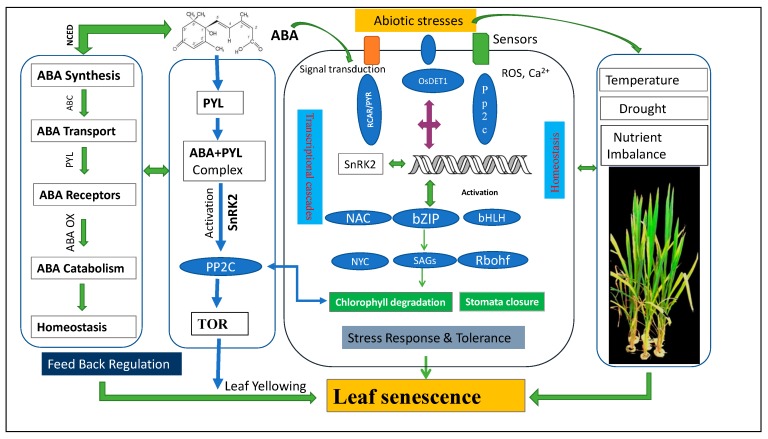
Systematic illustration of ABA-induced leaf senescence in response to abiotic stresses. *DET1*: *DE-ETIOLATED1*, ABC: ATP-binding cassette, TOR: target of rapamycin, bHLH: basic helix-loop-helix TF, Rbohf: respiratory burst oxidative homolog.

Receptor-like protein kinase 1 (RPK, a membrane-bound leucine-rich repeat receptor-like kinase) acts as an upstream component of ABA signaling. RPK is also a positive regulator of ABA. ABA receptors regulate SnRK2 activities in response to environmental stress. The receptors’ expression proportionally increases with ABA along with the progression of leaf senescence. Once the ABA concentration is increased in the cell, the concentration is sensed by the START domain containing PYL/RCAR components of ABA receptor gene family proteins [46,47]. ABA also binds to soluble receptor PYL proteins, forming the PYL–ABA complex, which inhibits protein PP2C (Figure 3). This interaction can be ABA-dependent or not [89,90,91,92,93,94]. The suppression of PP2C by ABA–PYL complex induces the repressing effect on SnRK2 by PP2C, leading to the auto-phosphorylation of SnRK2 [47]. The activated SnRK2 then induces the expression of ABRE-binding factors, b-ZIP TFs, and the activation/repression of ion channels/enzymes [89,95]. ABA-dependent gene expression induces the onset of leaf senescence in plants. The ABA signaling pathway has additional main constituents, such as PP2C, SAPK2, and OREB1, which have been identified in rice [96]. The crystal structure of the ABA–OsPYL–OsPP2C complex has been determined, and *OsPYL* has been further characterized through bioinformatics and biochemical analysis [97].

Promoters such as PP2C and SAG113 act as negative regulators of stomatal movement and water loss during leaf senescence. *SAG113* is expressed in senescent tissues, and it is induced by exogenous ABA application; *SAG113* knockout mutants delay leaf senescence [98,99]. Another investigation has hypothesized that *PYL9* activates SnRK2s to promote leaf senescence. Activated SnRK2s regulate *SAG12* expression, and PYL cannot activate SAG12-LUC expression in *SnRK2.2/3/6* triple-mutant protoplasts. Moreover, the SAG12-LUC expression is not enhanced by exogenous ABA treatment in *SnRK2.2/3/6* triple-mutant protoplasts, which can be recovered by the transfection of *SnRK2.6*, suggesting *PYL9* as a promoter of ABA-induced leaf senescence [52]. The ABA-activated SnRK2 is ethylene-independent, thereby phosphorylating ABFs and ABAI3/VP1 (RAV1) TFs. Then, the phosphorylated ABFs and RAV1 induce the expression of SAGs, especially upregulating *Oresara 1* (*ORE1*). *PYL9* knockdown mutants and ABA-insensitive, *PYL8*-*PYL9* double, and *SnRK2.2/3/6* triple mutants show an insensitive response toward ABA-induced leaf senescence, whereas *PYL9*-overexpressing mutants are sensitive to ABA-induced leaf senescence. Leaf senescence creates an osmotic gradient potential, allowing the flow of water in young and growing tissues, inducing the drought-resistant [52].

### 3.4. Involvement of Secondary Messenger Action Ca^2+^ in ABA Signaling and Leaf Senescence Regulation

ABA regulates leaf senescence through the signal transduction of Ca^2+^ secondary messenger action [54,100]. ABA deteriorates the membrane process to alter cell functions by activating the calcium signaling of cytosol. Calcium at low concentrations (0.1–1.0 μM) may block the onset of leaf senescence by increasing the hydraulic permeability and suppressing the loss of Chl and protein content. Applying Ca channel blocker in detached leaves induces senescence by the sharp reduction of Chl and expression of SAG and lipid peroxidation [100]. Calmodulin (CaM) delays leaf senescence by activating the transcript amount of senescence-related genes (Figure 2) [101].

Calcium delays leaf senescence, because CaM inhibits SAG expressions. CaM is a calcium-binding protein related to calcium-sensor proteins [102], leading the transduction of Ca^2+^ signals. CaM has four isoforms: CaM1/CaM4, CaM2/CaM3/CaM5, CaM6, and CaM7. They are encoded by seven genes (i.e., *CAM1*, *CAM2*, *CAM3*, … *CAM7*) in *Arabidopsis* in which *CaM1* encodes the Ca^2+^ binding protein CaM1. *CaM1*-overexpressed mutants display early leaf senescence by inducing ROS and expressing senescence-associated gene 12 (*SAG12*). ABA-induced ROS is reduced in amiRNA-*CaM1*, thereby delaying leaf senescence. Receptor protein kinase 1 (RPK1) in plasma membrane regulates ABA signaling, plant growth, stress signaling, and leaf aging [53,103,104,105]. RbohF is an NADPH oxidase (as described in Section 3.2) and contains EF-hand Ca^2+^-binding motifs referring to the regulatory effect of Ca^2+^ and phosphorylation on its capacity for ROS production [106]. Rbohf acts as a bridge between RPK1 and ROS production, along with SAG expressions [107]. The following three signaling components keep the positive-feedback regulation such that RPK1 regulates *CaM1* gene expression and CAM1 protein induces *RbohF* gene expression [108]. Expression analyses revealed that *CaM1* is positively regulated by RPK1, *RbohF* by CaM1, and *CaM1* by RbohD and RbohF, suggesting a positive-feedback loop among the three signaling components at a transcriptional level [108]. Calcium blocker treatment inhibits the elevation of cytosolic calcium ([Ca^2+^]cyt) in *EAS1-1* (novel ABA2 allelic mutants) in stresses. In guard cells, the Ca^2+^ channel activity is disrupted as the calcium gene is expressed. The calcium channel activity in the plasma membrane and the induced cytoplasmic concentration is suppressed in *EAS1*. When the calcium channel blocker is applied, the Chl and ion leakage of *EAS1* mutants are induced faster than those of its wild type. The mutation in *EAS1* inhibits [Ca^2+^]cyt elevation under multiple stresses. The ABA-activated Ca^2+^ channel activity is disrupted in EAS1 mutant by enhancing calcium channel gene expression. Therefore, elevated ABA-induced [Ca^2+^]cyt leads to early leaf senescence [54].

## 4. ABA Regulates TFs to Induce SAG Expressions

In plants, senescence is tightly controlled by senescence TFs, which mediate environmental and endogenous signals by modulating many SAG expressions [109,110,111]. The ultimate result suggests to induce SAG expressions, which are controlled by several senescence-promoting, plant-specific NAC TFs such as ORE1 [112], Oresara 1 sister 1 (ORS1) [113], and AtNAP [114].

### 4.1. Induction of NAC TFs

Plant proteins NACs are derived from three genes, *NAM* (No apical meristem), *ATAFs* (*Arabidopsis* transcription activation factor), and *CUC2* (cup-shaped cotyledons), which constitutes a wide range of plant-specific TFs [115]. NAC TFs have been reported to integrate with ABA signaling for the progression of leaf senescence in *Arabidopsis*, rice, and other plants [12,116,117,118,119]. A total of 151 and 117 *NAC* TFs have been reported in rice and *Arabidopsis thaliana*, respectively [120,121,122]. In *Arabidopsis*, AtNAP (ANAC029) [114] and ORE1 (ANAC092) [112,123] have been identified as central positive regulators of leaf senescence. NAC-like, activated by AP3/PI (NAP) regulates different ABA-induced leaf senescence in dicot and monocot species [124]. NAP in rice—ortholog of *AtNAP* (*OsNAP*)—and *Arabidopsis* (*AtNAP*) are located downstream of ABA biosynthesis and signaling. NAP in *Arabidopsis* promotes Chl degradation by inducing abscisic oxidase 3 (AAO3) to promote ABA-induced leaf senescence [125]. PP2C protein (described in Section 2.3) is positively regulated by *AtNAP*, which negatively regulate ABA-induced stomatal closure to trigger water loss as first sign of leaf senescence [98]. AtNAP binds to the promoter region of *AAO3* (controlling ABA production) to consequently enhance *AAO3* transcription and educe ABA production. This ABA accumulation enhances the transcription of key Chl degradation genes *STAY-GREEN1*, *NYC1*, pheophytinase, and *PAO* [125]. *OsNAP* also regulates ABA production, which induces SAG expressions [119] in rice. Similar to *AtNAP*, *OsNAC2* accelerates leaf senescence by activating ABA biosynthesis genes and Chl degradation [12]. PYL9 promotes leaf senescence by activating SnRK2s and inhibiting PP2Cs, which then phosphorylate ABA-insensitive 3/VP1 (RAV1) and ABF2 TF, consequently leading to the upregulation of ORE1 and other NAC TFs to target and induce SAG expressions [52].

Leaf senescence is delayed in *AtNAP* null mutants and promoted in the overexpressed *AtNAP* lines of *Arabidopsis* [126]. *AtNAP* binds to the promoter of a Golgi-localized PP2C (SAG113) and activates its expression [124]. *AtNAP* induces SAG113 expression, which inhibits stomatal closure to promote water loss and accelerate leaf senescence, whereas leaf senescence is delayed in knock-out lines [98], thus confirming *AtNAP* for the progression of leaf senescence. Similarly, the same inducing and delayed trend has been observed in rice, that is, the overexpression of *OsNAP* promotes leaf senescence and knocks down this gene-delayed senescence, indicating that *OsNAP/PS1*—a functional ortholog of *AtNAP*—mediates ABA-induced leaf senescence by direct transcriptional activation of several Chl degradation and SAGs, including *SGR*, *NYC1*, *NYC3*, *RCCR1*, *Osh36*, *OsI57*, and *Osh69*. *OsNAP* suppresses ABA biosynthesis-related genes, including *OsNCED1*, *OsNCED3*, and *OsNCED4*, thereby controlling ABA synthesis via a feedback mechanism [119]. In cotton (*Gossypium hirsutum*), the putative ortholog of *AtNAP* (i.e., *GhNAP*) has been identified as a positive regulator of ABA-mediated leaf senescence. Reduction in *GhNAP* expression results in delayed senescence and improved cotton yield and fiber quality [127].

In tomato, ABA-activated NAC TF named *SlNAP2,* which is the tomato putative ortholog of *AtNAP* from *Arabidopsis* and *OsNAP* from rice, plays a major role in regulating leaf senescence, which ultimately affects yield and fruit quality. Thus, *SlNAP2* is revealed to be a positive regulator of leaf senescence, and the senescence regulatory module controlled by *SlNAP2* is conserved between tomato and other plant species whose NAP-control mechanisms have been characterized. *SlNAP2* directly controls the expression of the senescence-associated gene *SlSAG113*, a homolog of *Arabidopsis SAG113*, and Chl degradation-related genes *SlSGR1* and *SlPAO*. Intriguingly, *SlNAP2* directly regulates the expression of ABA biosynthesis (*SlNCED1*) and ABA degradation (*SlCYP707A2*) genes, suggesting the existence of a “self-regulating” mechanism by which SlNAP2 tunes its dynamic expression in leaves [124].

Transgenic lines with reduced expression of *SlNAP2* exhibit a significant delay in leaf senescence, along with an increase in fruit yield and fruit sugar content. Therefore, leaf senescence regulation is important to achieve increased fleshy fruit yield and sugar content. ABA-induced senescence is also significantly impeded in *SlNAP2*-suppressed plants, suggesting an important role of *SlNAP2* in ABA-induced leaf senescence. Furthermore, NACs are the important leaf senescence regulators in relation to environmental stresses and ABA. The TF of phytochrome factor 4 (*PIF4*) and *PIF5* induces OREI in dark-induced senescence [128]. The mechanism for the ABA-induced upregulation of *AtNAP*, *OREI*, and *OsNAP* is still unknown.

### 4.2. Modulating OsNAC2 TF Expression

Mao et al. [12] reported that ABA also integrates *OsNAC2* to regulate leaf senescence by upregulating the target SAGs *OsSGR* and *OsNYC3*. The exogenous ABA application to OsNAC2-OX lines significantly reduces the shoot length and leaf yellowing, with a further Chl degradation trend. The application also increases the ABA content. The opposite trend is observed in RNAi lines, suggesting that ABA positively regulates *OsNAC2* expression. The increased ABA content in OsNAC2-OX is due to the upregulation of ABA anabolism genes *OsNCED2*, *OsNCED3*, *OsNCED5*, and *OsZEP1* with the significant downregulation of ABA catabolism genes *OsABA8ox1*, *OsABA8ox2*, and *OsABA8ox3*, conferring *OsNAC2* regulation for ABA anabolism and catabolism. Thus, the activation of Chl degradation genes along with ABA biosynthesis genes is also regulated by NAC2. *OsNAC2* increases ABA concentration by inducing the expression of ABA anabolism genes such as *OsNCED3* and *OsZEP1*, and downregulating ABA catabolism gene *OsABA8ox1*. ABA endogenous concentration controls *OSNAC2* expression by a feedback mechanism. In addition, repression-like *OsNAC2* expression is downregulated at high ABA concentration and low ABA concentration, directly inducing its expression. Moreover, *OSNAC2* induces the expression of the Chl degradation genes *OsSGR*, and *OsNYC3*. Interestingly, the reduced *OsNAC2* expression leads to a 10-fold increase in the yield of RNAi *NAC2* lines. The regulatory model of ABA–NAC–SAGs elucidates the transcriptional network of ABA signaling and production, which can intervene ABA–NAC–SAG interaction with respect to other regulatory factors [12].

### 4.3. Regulation of bZIP TF

*OsbZIP* mediates abiotic stresses by interacting with *SAPK2* and *OsPP2C49*, an *ABI-insensitive 1* (*ABI1*) homolog for its transcription activation [129]. Among them, *OsbZIP23* is involved in the function of stress-responsive genes and hormone signaling and aging development process by positively regulating *OsPP2C49* to decrease the sensitivity of ABA response and rapid dehydration. *OsbZIP23* also positively regulates the *OsNCED4* in ABA biosynthesis (as discussed in Section 2.1). Overexpressed *OsbZIP23* mutants express an increased ABA sensitivity, thus improving drought tolerance, leaf senescence, and salt tolerance [130]. *OsbZIP46* (ABL1) has a high sequence identity to *ABF/AREB* TFs, *ABI5*, and *OsbZIP23*. Similarly, *OsbZIP46*-overexpressing mutants are ABA sensitive, but drought and salt tolerance are comparatively lower than *OsbZIP23*. The overexpression of *OsbZIP46CA1*, an active form of *OsbZIP46*, induces the activation of downstream genes to increase drought tolerance [131]. SAPK2 can directly phosphorylate and activate *OsbZIP23*, whereas *OsPP2C49* negatively regulates *SAPK2* and further inhibits the transcription activity of *OsbZIP23* [129].

In several studies based on ABA-deficient-2 alleles (*ABA2*), *ABA1*, *ABA3*, and *AAO3*, *NCED3* has been isolated in *Arabidopsis*-screening mutants [33,132]. These mutants have clearly shown the role of ABA in stomatal regulation, development processes, and stress responses. In *Arabidopsis*, NAC-like, activated by AP3/PI (NAP) TF enhances *AAO3* transcription, thus improving ABA levels and promoting Chl degradation [125]. Interestingly, Park et al. [1] reported that MYB-related TF *O. sativa* RADIALIS-LIKE3 (*OsRL3*) is involved in dark-induced leaf senescence in rice as mediated by ABA. The *OsRL3* knockdown mutant OSRl3 is slightly sensitive to ABA. Moreover, salt stress shows stay-green phenotype. Another leucine zipper TF, ABI5, mediates ABA and stress-induced signaling, which includes PYR/PYL/RCAR receptors, PP2C phosphatases, and SnRK2 kinases to regulate ABRE genes motif within their promoter region (Figure 3) [133].

## 5. ABA Regulation of Senescence-Related Membrane-Associated Protein to Transduce Leaf Senescence

The *senescence-associated secretory phenotype* (*SASP*) encodes subtilases (SBTs), which regulate ABA signaling to induce leaf senescence [134]. The expression of subtilases is induced by darkness, ABA, and ethylene treatments. *SASP* knockout mutants are sensitive to ABA in seed germination and seedling growth. The ABA-induced leaf senescence in such mutants is stronger, and their ROS production is higher than those in the wild type. Exogenous ABA concentration alters the expression pattern of six ABA signaling-related genes (i.e., *ABI1*, *HAB1*, *RBOHD*, *RBOHF*, *PYL4*, and *OST1*) to consequently increase drought tolerance. SASP physically interacts with open stomata 1 (*OST 1*) at the cell periphery level [134]. In addition, the expression of *OST1* and *SASP* induces the degradation of OST1 in controls. However, in *sasp-1* protoplasts, the co-expression of *OST1* and *SASP* does not lead to the degradation of OST1 along with the decreased attenuation rate of guard cells. SBTs control the plant-specific developmental process. In *Arabidopsis*, senescence-associated subtilisin protease (SASP, AtSBT1.4) has been reported to regulate ABA during leaf senescence [134,135]. SASP is induced by the application of ABA and darkness, suggesting that SASP is also related to sugar starvation. Furthermore, *SASP* knockout mutants (*SASP-1*) show sensitivity to ABA, with increased leaf senescence and increased ROS production. SASP leads the OST1 degradation by first interacting with OST1 at the cell periphery level, opening another gateway of senescence where SASP regulates ABA by interacting with OST1 and inducing OST1 degradation. In another investigation, the U-box senescence-associated E3 ubiquitin ligase 1 (SAUL1) was reported to regulate ABA biosynthesis and signaling. It interacts with AAO3, which is a key enzyme for ABA biosynthesis, to start protein degradation. The increased amount of AAO3 induces increases in the level of ABA in SAUL1 mutants with increased leaf senescence [136]. ABI1 is a kind of phosphatase that inhibits OST1 activity in the absence of ABA. ABI1 is targeted and ubiquitinated by U-box E3 ligases PLANT U-BOX 12 (PUB12) and PUB13 for degradation after ABA-bound PYR1 interacts with ABI1, leading to ABI1 degradation by the 26S proteasome [137].

### ABA mediates the Target of Rapamycin (TOR) to Induce Leaf Senescence

TOR is a potential regulating bridge between stress response and growth. TOR regulates protein synthesis, cell size, growth and development, transcription, and metabolism in plants [138,139,140]. For protein synthesis, TOR initiates the phosphorylation of E2Fa TF and 40S ribosomal protein S6 kinases (S6Ks) [140]. TOR also regulates BR signaling by inducing the expression of BR signaling key TF BZR1 to mediate the effect of sugar signaling, which attributes the abiotic stress response to leaf senescence [141]. Wang et al. [142] reported that TOR kinase phosphorylates the PYL–ABA receptor in normal growth conditions. TOR kinase also restricts ABA to interact with downstream PP2C enzymes, conferring this negative regulation as the best fit for ABA-independent PYLs signaling under optimum conditions.

Under stress, ABA inhibits TOR activity by inducing the PYL-mediated activation of SnRK2s (as described above), which phosphorylates the TOR regulator Raptor and is called ABA-induced stress growth inhibition. The growth recovers when PYLs are phosphorylated, forming a complex phosphorylation-dependent regulatory loop between ABA and TOR complex to repress stresses and ABA responses under unstressed conditions. Thus, growth under stress is inhibited, and growth recovery is promoted once environmental stresses subside.

## 6. Gene Mutation as the Key Step to Identify ABA Regulation for Senescence

Exogenous ABA induces early senescence. Endogenous ABA accumulates in response to abiotic stresses, leading to the expression of several TFs and SAGs. More than 132 SAGs have been identified on the 12 chromosomes of rice in the updated leaf senescence database (http://psd.cbi.pku.edu.cn/). Song et al. [54] isolated two *Arabidopsis* mutants (*EAS1-1* and *EAS1-2*, novel allele of ABA2) that show early senescence, insensitivity to stress, and large stomatal aperture (Table 1). SAGs are upregulated in the early stage of development. In addition, calcium channel activities in guard cells are very low. These studies can correlate the role of ABA in the regulation and onset of leaf senescence. The map-based cloning of *EAS1-1* and *EAS1-2* revealed missense mutations in the second exon of *At1g52340*, in which the 190 glutamic acid and the 265 glycine are replaced with lysine and arginine, respectively. These genes are the allelic forms of *ABA2*/*GIN1*/*SRE1* that encode short-chain dehydrogenase/reductase (SDR1), which catalyzes the oxidation during the conversion of ABA aldehyde (ABAld) [33,143]. This mutation dramatically increases SAG expression (*SAG29*, *12*, *13*, *14*, *101*, *113*, *25*) by many folds, especially *SAG12*, which expresses a 1500-fold higher expression compared to SAG29, 21, and 113, because of the deletion of oxidation-related genes for *ABAl*. 

## 7. Integrated Crosstalk that Initiates the Onset of Leaf Senescence

### 7.1. Interaction of Sugar Signaling with ABA during Leaf Senescence

Sugar coordinates with ABA to induce leaf senescence, and it works as an interacting bridge between sugar signal and leaf senescence. ABA mutants are insensitive to sugar level. Similarly, ABAI and ABA-deficient mutants do not respond in sugar alteration signal in *Arabidopsis* [146]. However, the investigated works on sugar-insensitive mutants were focused on the seedling stage, and little is known about its effects on senescence at the mature growth stage. Sugar has a solid relation with nitrogen allocation, as it coordinates nitrogen availability [147]. Glucose (GLC), fructose (FRU), and sucrose (SUC) are proven to induce leaf senescence in the presence of ABA and ethylene, suggesting that development factors (mostly ABA) act as bridges to induce leaf senescence [148]. There is controversy regarding sugar signaling, as to whether sugar starvation induces leaf senescence or sugar accumulation. Several findings support both hypotheses and are still under debate. Sucrose/hexose ratio mediates cell wall invertase activity, which delays leaf senescence in tomato and tobacco [149,150]. *HXK1* is involved in sugar signaling to mediate leaf senescence as *HXK1* overexpresses tomato and *Arabidopsis* plants, thus accelerating leaf senescence in the presence of ABA [151]. In addition, *HXK1* knockdown mutants have shown delayed symptoms of leaf senescence, but concrete *HXK1* metabolic pathways must be fully elaborated. T6P protein is involved in sugar-induced leaf senescence. Moreover, transgenic plants with low T6P level express sugar insensitivity with delayed leaf senescence symptoms [148]. However, few investigations have stated the temporal effect of T6P, and at the late development stage, leaf senescence is independent of T6P response [148,152]. Similarly, T6P inhibits SnRK1 in the presence of additional factors [148,153]. If T6P acts via SnRK1, then T6P initiates senescence before symptoms become visible. This condition is supported by the notion that T6P is required for early developmental changes that result in the competence to respond to other senescence-inducing factors, such as GLC or ethylene [153].

The carbon/nitrogen (C/N) signaling mechanism controls the growth and metabolism of plants. C–N insensitive mutants (CNI2-D) can overcome the post-germination growth with normal green cotyledons in low N and high C stress conditions [154]. ABA acts as a bridge between C/N signaling and growth metabolism by its central signaling transduction component ABI1 [155]. In *Arabidopsis*, the overexpressed ABI1 is insensitive to C/N stress, whereas the knockdown mutant ABI1-2 is hypersensitive to ABA. C/N stress alleviates the expression pattern of *SnRK2s* and *SnRK1s* genes, whereas *ABI1* significantly suppresses the expression of SnRKs in the following stress. The *CNI-2* gene encodes phosphate protein ABI, which negatively regulates ABA signaling [155]. By contrast, the *CNI-1* gene encodes ubiquitin ligase ATL31, and overexpressed *ATL31* plants persist in normal growth at low N and high C [156].

### 7.2. Interacted Crosstalk between Ethylene and ABA

Zhao et al. [52] reported that ABA-induced leaf senescence is independent of ethylene. First, they stated that ethylene also induces leaf senescence in an experiment involving protoplast treatment with the ethylene biosynthesis inhibitor aminoethoxyvinylglycine (AVG), which suppresses the expression of SAG12-LUC in the absence of ABA. However, AVG does not inhibit the ABA-induced (PYL9-enhanced) *SAG12-LUC* expression. They generated ethylene-resistant mutants e*in2-1* and e*in3-1*, which are insensitive (i.e., no effect) to ABA-induced leaf senescence. Moreover, e*in2-1* mutants do not block ABA-induced and PYL9-enhanced *SAG12-LUC* expression. Induction is addressed by the generation of *PYL9* mutant tagged with HA and YFP under the native promoter (*ProPYL9:PYL9-HA-YFP*) to further confirm the ethylene-independent ABA pathway of leaf senescence. They isolated *PYL9*-induced PP2C proteins such as HAB2, PP2CA, and ABI1 in an ABA- or osmotic stress-enhanced manner. PYL9 interacts with all PP2Cs tested in an ABA-in- dependent manner in yeast two-hybrid (Y2H) assays. They fused the 788 bp fragment of the *SAG12* promoter (*SAG12-LUC*) to the LUC reporter gene and used the construct as a senescence-responsive reporter. The 788 bp *SAG12* promoter contains the 9-mer sequence T(TAG)(GA)CGT(GA)(TCA)(TAG).

## 8. Conclusions and Future Recommendations

As the dominating phytohormone, ABA regulates the internal metabolic functions to alleviate the harms of environmental stresses. Leaf senescence is a temporal niche with integrated aging and phytohormone regulatory factors. The dominating environmental stresses intervene with phytohormone regulatory factors to trigger and/or accelerate leaf senescence, which is a limiting factor of quantitative and qualitative traits. From the regulation of physiological processes during leaf senescence, and pre-transcriptional changes, to post transcriptional alteration, the ABA working pattern is controlled by a network of ABA biosynthesis, ABA transport, and ABA signaling receptors, and ABA catabolism depends upon ABA biosynthesis. The major regulators of ABA biosynthesis, ABA transport, receptors, and ABA catabolism are NCED, PYL, MRP, and CYP families, respectively. ABA regulates NYCs and D1 protein to start Chl degradation and destruction as the first sign to limit, reduce, or terminate photosynthesis. ABA integrates with antioxidant responses and triggers the pre-transcriptional induction of NOXs to modulate NADPH oxidase for ROS generation. In addition to an increase in H_2_O_2_, SOD, APOD, GR, and CAT, a decrease in AsA and GSH contents is observed in rice leaves.

The regulatory network of ABA includes SnRK2 kinases, RCAR, and PP2Cs, and PP2C suppression by an ABA–PYL complex induces the repressing effect on SnRK2 by PP2C, leading to the auto-phosphorylation of SnRK2. The activated SnRK2 then induces the expression of ABRE-binding factors and bZIP TFs and the activation/repression of ion channels/enzymes. ABA deteriorates the membrane process to alter cell functions by activating calcium signaling to regulate secondary messenger action, which increases Chl loss, SAG induction, lipid peroxidation, and CaM activation. ABA tightly regulates senescence TFs, which mediate environmental and endogenous signals by modulating many SAG expressions. The important TFs are NYCs, NACs, and bZIPs. ABA regulates BR signaling by inducing BZR1 expression to mediate the effect of sugar signaling, which attributes the abiotic stress response to leaf senescence.

Mutants for defects in ABA synthesis and stomatal response provide effective tools to determine the relationship between stomatal behavior and senescence onset, which alters stomata opening, blue signal release, and ion transport activity. These factors then alter the mechanism of leaf senescence. Therefore, understanding the functional regulation of ABA-imparting leaf senescence is necessary to ensure food security in most populous regions of the world. Understanding ABA’s functions provides foundations for the engineering of new orthogonal ABA receptors, designing agrochemicals against functional ABA receptors, and novel genetic engineering strategies for enhancing abiotic stress tolerance, inducing senescence and yield. The functional role of rice PYLs in ABA-mediated stress response should also be elucidated to engineer rice and other related cereal crops for improved and delayed senescence capability under abiotic stresses. The importance of repairing PSII activity through the re-synthesis of the D1 protein during light exposure is demonstrated by an increase in the recovered amount of D1 protein in darkness, accompanied by a decrease in ABA concentration that should be further elaborated with gene modulation and qTL mapping-based studies. The molecular patterns of NOX isoforms implicated in ROS generation in response to ABA should also be further investigated in detail for the induction of leaf senescence. PIF4 and PIF5 TFs induce OREI in dark-induced senescence. The mechanism of the ABA-induced upregulation of *AtNAP*, *OREI*, and *OsNAP* should also be elaborated.

## Figures and Tables

**Table 1:** Mutations that alter the effects of ABA-induced leaf senescence.

S. No	Genotype	Mutation	Treatment	Transgene	Effect	Reference
1	*Arabidopsis*	−	Cold 4 °C, 400 mM NaCl, 500 mM mannitol, 10 mM H_2_O_2_ in 7-day-old plants	*eas1-1*	Accelerated leaf senescence by reducing Ca^2+^ concentration	[54]
2	Rice	EMS	Darkness and 200 µM exogenous ABA for 5 days	*psl15*, *psl50*, *psl85, psl89*, *psl117* and *psl270*	Induced early and premature leaf senescence, increased malondialdehyde content	[80]
3	Rice	+		nyc1		[57]
4	Rice	+	5 d dark, exogenous ABA in detached leaves (4 µM)	*OsNAC2*-OX	Induce leaf senescence (4-week-old + grain filling)	[12]
5	Rice	−	5-d dark treatment, exogenous ABA in detached leaves (4 µM)	*OsNAC2*-RNAi18	Delayed senescence (4-week-old + grain filling)	[12]
6	Rice	+		*ps1-D*	Promoted premature leaf senescence	[119]
7	Rice	−		*OsNAP*	Delayed leaf senescence	[119]
8	Rice	EMS	10 days dark treatment in detached leaves, exogenous ABA to detached leaves after 10 day of flowering	*bml*	Promoted leaf senescence by reducing chlorophyll contents	[22]
9	*Arabidopsis*	+	Drought/exogenous 100 µM ABA	pRD29A:*PYL9*	Induced resistance to drought and accelerated ABA-induced leaf senescence	[52]
10	*Arabidopsis*	+	Exogenous 100 µM ABA in 4-week-old detached leaves	*abf2abf3abf4* (triple mutant	Delayed leaf senescence by blocking signal for chlorophyll degradation	[60]
11	*Arabidopsis*	+	Exogenous 100 µM ABA in 4-week-old detached leaves	*snrk2.2/2.3/2.6* (Triple mutant)	Inhibited chlorophyll degradation with stay-green phenotype	[60]
12	*Arabidopsis*	+	Exogenous 100 µM ABA in 4-week-old detached leaves	acd1-20, *nyc1*-1	Inhibited signaling in Chl and LHC (light harvesting complex) degradation pathways	[60]
13	*Arabidopsis*	+	Exogenous 50 µM ABA in 3-week-old detached leaves	*CaM1*	Triggered the accumulation of ROS and *SAG12* expression	[108]
14	*Arabidopsis*	−	Exogenous 50 µM ABA in 3-week-old detached leaves	*amiRNA-CaM1*	Delayed leaf senescence	[108]
15	*Arabidopsis*	−	100 µM ABA of 20 DAG leaves for 20 h	*SAG113*	Exhibited delayed leaf senescence	[99]
16	*Arabidopsis*	+	Drought stress at 18 DAG, dark-induced treatment at 30 DAG for 7 days	*OxMYBR1*	Delayed leaf senescence with strong holding capacity	[144]
17	*Arabidopsis*	−	Drought stress at 18 DAG, dark-induced treatment at 30 DAG for 7 days	*mybr1*	Reduced water loss, more rapid chlorophyll loss, and induced leaf senescence	[144]
18	Rice	−	3 μM exogenous ABA on 4-week-old detached leaves	*osrl3*	Showed ABA insensitivity and stay-green phenotype	[1]
19	*Arabidopsis*	−	50 μM exogenous ABA for 3 days	Sasp	Enhanced leaf senescence by increasing *SAG12* expression and ROS production	[136]
20	*Arabidopsis*	−	50 μm exogenous ABA for 8 days after 10 DAG	*clf-50 swn-1*	Induced leaf senescence	[145]

The senescence-induced mechanism with respect to the regulation of phytohormones and gene silencing and overexpressing strategy (where + indicates overexpression and − indicates down regulation. DAG: days after germination; EMS: ethyl methyl sulfonate.

## Abbreviations

ABA	Abscisic acid
PYL	Pyrabactin resistance 1-like
SnRK2	Sucrose nonfermenting 1-related protein kinase 2
ABFs	ABA-responsive element-binding factors
SAPK	Stress-activated protein kinase
PPH	Pheophytinase
SGR	Stay green
NADPH	Nicotinamide adenine dinucleotide phosphate
MDA	Malondialdehyde
PAO	Polyamine oxidases
NYC1	Non yellow coloring 1
SASP	Senescence associated secretory phenotype
PP2C	Protein phosphatase 2C
AAO3	*Arabidopsis* aldehyde oxidase 3
OST1	Open stomata 1
BZR1	Brassinazole-resistant 1
ABA2	Abscisic acid deficient 2
SRE1	Salt resistance 1
GIN1	Glucose insensitivity 1
T6P	Trehalose-6-phosphate
HXK1	Hexokinase 1
SUC	Sucrose
FRU	Fructose
GLC	Glucose
YFP	Yellow fluorescent protein
SAGs	Senescence-associated genes
EIN	Ethylene resistant mutant
LUC	Luciferase
AVG	Aminoethoxyvinylglycine
DET1	DE-ETIOLATED1
